# The role of acid inhibition in *Helicobacter pylori* eradication

**DOI:** 10.12688/f1000research.8598.1

**Published:** 2016-07-19

**Authors:** David R. Scott, George Sachs, Elizabeth A. Marcus

**Affiliations:** 1 Department of Physiology, David Greffen School of Medicine at UCLA, Los Angeles, CA, 90095, USA; 2 Department of Medicine, David Greffen School of Medicine at UCLA, Los Angeles, CA, 90095, USA; 3 Department of Pediatrics, David Greffen School of Medicine at UCLA, Los Angeles, CA, 90095, USA; 4 VA Greater Los Angeles Healthcare System, Los Angeles, CA, 90073, USA

**Keywords:** Helicobacter pylori, gastritis, gastric, pathogen, stomach, infection

## Abstract

Infection of the stomach by the gastric pathogen *Helicobacter pylori* results in chronic active gastritis and leads to the development of gastric and duodenal ulcer disease and gastric adenocarcinoma. Eradication of *H. pylori* infection improves or resolves the associated pathology. Current treatments of *H. pylori* infection rely on acid suppression in combination with at least two antibiotics. The role of acid suppression in eradication therapy has been variously attributed to antibacterial activity of proton pump inhibitors directly or through inhibition of urease activity or increased stability and activity of antibiotics. Here we discuss the effect of acid suppression on enhanced replicative capacity of *H. pylori* to permit the bactericidal activity of growth-dependent antibiotics. The future of eradication therapy will rely on improvement of acid inhibition along with current antibiotics or the development of novel compounds targeting the organism’s ability to survive in acid.

## Introduction

Infection of the stomach by the gastric pathogen *Helicobacter pylori* results in chronic active gastritis and leads to the development of gastric and duodenal ulcer disease, gastric adenocarcinoma, or mucosa-associated lymphoid tissue (MALT) lymphoma. Eradication of *H. pylori* infection improves or resolves the associated pathology, leads to ulcer healing rates of >90%, and is effective in preventing the recurrence of bleeding ^1– 3^. Low-grade MALT lymphoma can be treated by eradication of *H. pylori* ^4– 6^. However, success rates of standard therapy regimens, typically including acid suppression and multiple antibiotics, have fallen below the acceptable level of 80% in many parts of the world ^7^. The reason for the decline in treatment success is multifactorial, involving issues such as antibiotic resistance, patient compliance, level of acid suppression, and host and bacterial factors that alter the efficacy of treatment ^8^.

Contemporary therapies for the treatment of *H. pylori* infection rely on acid suppression in combination with at least two antibiotics. Inhibition of the gastric H ^+^,K ^+^-ATPase, the final step in the acid secretion pathway, by proton pump inhibitors (PPIs) was initially included in eradication regimens to aid in symptom relief in patients suffering from peptic ulcer disease. However, it was found that PPIs and antibiotics act synergistically in eradicating *H. pylori* and acid inhibitors have been included in successful eradication treatments ever since. This raises the question of why inhibition of acid secretion is required. One suggestion is that PPIs have antibacterial properties. *In vitro*, the PPIs lansoprazole and omeprazole were shown to have antimicrobial properties that were unique to *H. pylori* as determined by the agar dilution method at neutral pH ^9^. However, the antibacterial effect was seen only at industrial PPI concentrations (100 μg/ml) and only after 24 hours. The antibacterial effect of lansoprazole was attributed uniquely to the inhibition of *H. pylori* urease ^10^. However, it was later shown that the *in vitro* effect of PPIs on the reduction in *H. pylori* survival was independent of urease activity and reduction in survival in the presence of PPIs was not unique to *H. pylori* ^11^. Therefore, these findings, taken together, indicate it is unlikely that direct killing of the bacterium by PPIs contributes to the *in vivo* mechanism of PPI/antibiotic synergy.

Another possible mechanism for PPI/antibiotic synergy in *H. pylori* eradication is that antibiotics are more stable and have higher activity at higher gastric pHs afforded by PPIs. *In vitro*, the antibacterial activity against *H. pylori* of both the macrolide and the quinolone antibiotics was inversely proportional to pH ^12^. The MIC _90_ for the beta-lactam ampicillin was 2 μg/ml, 0.5 μg/ml, and 0.25 μg/ml at pH 5.7–6.0, pH 7.4, and pH 7.8–8.0, respectively. On the other hand, the antibacterial activity against *H. pylori* by metronidazole was independent of pH down to at least pH 5.7.

**Figure 1.**
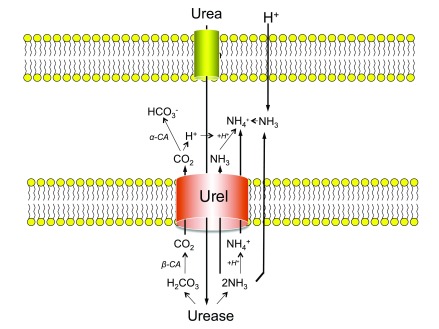
Acid acclimation by *Helicobacter pylori*. As the gastric lumen acidifies, the periplasmic pH of the Gram-negative pathogen *H. pylori* drops as well. Periplasmic acidification results in the protonation and opening of UreI, the proton gated urea channel in the inner membrane. With the opening of UreI, urea moves into the cytoplasm where it is hydrolyzed by urease, resulting in the production of carbonic acid and ammonia. Carbonic acid is converted to CO _2_ by the cytoplasmic β-carbonic anhydrase (β-CA) enzyme. The two gasses diffuse through the membrane and through UreI into the periplasm, where CO _2_ is converted to bicarbonate by the periplasmic α-carbonic anhydrase (α-CA) enzyme. The periplasm is then buffered to a pH of 6.1, well within the pH range of survival for a neutralophile.

## Acid acclimation

*H. pylori* colonizes an acidic niche in the human stomach ^13^. However, *H. pylori* is bioenergetically a neutralophile, able to survive at pH 4–8 and grow at pH 6–8 ^14^ and is slow growing, with a doubling time of 4–6 hours, in contrast to 20 minutes for *Escherichia coli*. *H. pylori* is able to not only survive but also flourish in the highly acidic gastric environment through the mechanism of acid acclimation. The main feature of acid acclimation is the ability to maintain periplasmic pH close to neutrality in the presence of acid ^15^. Acid acclimation is distinct from the acid resistance mechanisms of other bacteria that moderately elevate cytoplasmic pH to maintain viability but not growth in acid ^16^. Acid acclimation by *H. pylori* is dependent on urease, a neutral-pH-optimum cytoplasmic-localized enzyme, and a proton gated urea channel, UreI ^15, 17, 18^. Additionally, a periplasmic-localized α-carbonic anhydrase enzyme also contributes to periplasmic buffering by catalyzing the conversion of carbon dioxide produced by urease to bicarbonate ^15^.

An understanding of acid acclimation and *H. pylori* bioenergetics is critical to successful treatment protocols. *H. pylori* is uniquely adapted to survive in the acidic environment of the stomach; however, since it is a neutralophile, only a small fraction of the bacteria will actually be dividing or growing in acid as compared to more neutral pH. This is demonstrated in transcriptomal studies, where cell division and cell wall synthesis genes increase transcription at neutral pH as compared to acidic pH (pH 4.5) ^19^. The antibiotics amoxicillin and clarithromycin used for the eradication of *H. pylori* infection are dependent on bacterial growth and cell envelope synthesis and therefore will only be bactericidal to actively dividing bacteria ^19^. Since *H. pylori* that are not dividing at the time of antibiotic administration will not be killed by the antibiotics, the small population of viable bacteria restore colonization of the stomach once the antibiotics are stopped.

It is likely, therefore, that elevation of gastric pH by acid blockers stimulates *H. pylori* growth and accounts for their synergism with antibiotics. However, the median intragastric pH achieved by PPIs, at recommended doses, does not achieve the sustained pH elevation required to mimic the bactericidal effect seen in *in vitro* studies ^19, 20^. More effective inhibition of acid secretion would likely increase eradication rates with a single antibiotic such as amoxicillin, to which current resistance is <1% in Europe ^21^. However, amoxicillin resistance appears to be on the rise in Asia and Africa, so the antibiotic used for dual therapy should be based on geographical resistance rates ^22, 23^. Support for this concept is found in eradication studies of high-dose dual therapy (HDDT), slow PPI metabolizers, and novel potent acid inhibitors.

Various dual therapy eradication regimens with PPIs and amoxicillin have been studied for over 20 years, but consistency in results has not been established. Meta-analysis of dual therapy with omeprazole 20 mg *bis in die* and amoxicillin >2 g total daily suggested a >80% eradication rate and hinted at the importance of acid suppression in these regimens. However eradication rates of 30–50% were seen with similar dual regimens in other studies ^24, 25^. In one study, it was shown that the time with pH above 4 and continuous periods with intragastric pH above 6 were significantly associated with successful treatment of *H. pylori,* indicating that profound acid suppression was responsible for improved eradication rates. Amoxicillin dosing frequency is also an important factor as well. Amoxicillin, unlike many other antibiotics used in *H. pylori* eradication regimens, is time dependent, not concentration dependent, so time above MIC _50_ is an important factor in efficacy ^26, 27^. A regimen of four times daily rabeprazole (20 mg) and four times daily amoxicillin (750 mg) for 14 days was superior to standard triple therapy, with eradication greater than 90% ^28^.

More evidence that profound acid inhibition is efficacious in *H. pylori* eradication comes from studies of PPI metabolism. PPIs are metabolized in the liver by cytochrome CYP2C19 ^29^, polymorphisms of which affect the pharmacokinetics and pharmacodynamics of the medication. The phenotypes of the CYP2C19 polymorphisms are rapid metabolizers (RM), intermediate metabolizers (IM), and poor metabolizers (PM) ^30^. The plasma ‘dwell time’ of a single dose of omeprazole is directly correlated with the rate of metabolism among the different alleles with PM>IM>RM, which in turn affects the level of acid inhibition, again with PM>IM>RM. The difference in the rate of metabolism influences the efficacy of *H. pylori* eradication, since the rate of eradication correlates well with the degree of acid inhibition; however, genotype is less of a factor as PPI doses are increased ^31^. A standard 20 mg dose of omeprazole in poor metabolizers produced >90% eradication ^28^.

## PCABS: Potent acid inhibitors

An alternative to high-dose PPI for eradication of *H. pylori* is the development of more potent acid suppressive agents. For example, vonoprazan, a potassium competitive acid blocker (PCAB), has the benefit of a more rapid and sustained acid inhibitory effect regardless of CYP2C19 genotype ^32^. Vonoprazan is a weak base (pK _a_ 9.06) that accumulates in the parietal cell canaliculi at concentrations of up to 10 ^8^ fold higher than in the blood (pH 7.4) ^33^. This inhibitor is highly selective for the gastric H ^+^,K ^+^-ATPase with a K _i_ of 10 nM and an IC _50_ of 17–19 nM ^33^. Binding is rapid, very slowly reversible, and reaches a plateau of inhibition within 200 seconds. The *t*_1/2 _of dissociation is 4.7 and 7.5 hours for rabbit and hog proton pumps, respectively ^33^. Taken together, these parameters indicate that vonoprazan has the potential for rapid, reversible, and long-lasting inhibition of acid secretion when compared to the PPIs. The unique properties of vonoprazan combined with its greater degree of acid inhibition as compared with the PPIs, should provide greater efficacy in *H. pylori* eradication. Use of vonoprazan in place of PPIs has shown early promise in recent studies of Japanese populations, with an eradication rate of 70.2% using vonoprazan, amoxicillin, and clarithromycin as second-line therapy in patients who have failed first-line treatment with rabeprazole, amoxicillin, and clarithromycin and a 92.7% success rate of first-line therapy with vonoprazan, amoxicillin, and clarithromycin ^34, 35^.

## Conclusion

*H. pylori,* being a neutralophile, replicates best at neutral pH. The requirement for acid inhibition in *H. pylori* eradication therapy is to raise the pH at the site of infection to allow growth of the organism and increase the efficacy of growth-dependent antibiotics. Increasing acid suppression by using higher doses of currently available PPIs or via development of novel acid inhibitors such as the PCAB vonoprazan or the use of antibiotics with low rates of antibacterial resistance will improve the rate of *H. pylori* eradication. The future of eradication therapy will depend on improvement of acid inhibition with current antibiotics or perhaps a totally new class of antibiotics. An exciting alternative, specific to *H. pylori*, would be compounds targeted to essential acid acclimation proteins such as UreI or carbonic anhydrase.
